# Relationship Between Mortality and Seizures After Intracerebral Hemorrhage: A Systematic Review and Meta-Analysis

**DOI:** 10.3389/fneur.2022.922677

**Published:** 2022-06-20

**Authors:** Hong-yu Lin, Qing-qing Wei, Jian-yi Huang, Xing-hua Pan, Ning-chao Liang, Cai-xia Huang, Teng Long, Wen Gao, Sheng-liang Shi

**Affiliations:** ^1^Department of Neurology, People's Hospital of Chongzuo City, Chongzuo, China; ^2^Department of Neurology, People's Hospital of Liuzhou City, Liuzhou, China; ^3^Department of Neurology, Second Affiliated Hospital of Guangxi Medical University, Nanning, China

**Keywords:** intracerebral hemorrhage, outcome, mortality, seizures, systematic review, meta-analysis

## Abstract

**Background:**

The relationship between mortality and seizures after intracerebral hemorrhage (ICH) has not yet been understood until now. A meta-analysis was performed to assess the effect of post-ICH seizures on mortality among patients with ICH.

**Methods:**

PubMed and Embase were searched from the establishment of the databases to December 2021 to identify literature that evaluated the relationship between post-ICH seizures and mortality in ICH. Crude odds ratios and adjusted odds ratios with a 95% confidence interval (CI) were pooled using a random-effects model.

**Results:**

Thirteen studies involving 245,908 participants were eventually included for analysis. The pooled estimate suggested that post-ICH seizures were not associated with significantly increased mortality in patients with ICH (crude odds ratios 1.35, 95% CI: 0.91–2; adjusted adds ratios 1.22, 95% CI: 0.78–1.88). However, the relationship was not consistent in subgroup analysis or robust in a sensitivity analysis.

**Conclusions:**

This meta-analysis proved that post-ICH seizures were not associated with significantly increased mortality in patients with ICH. However, this result could be influenced by confounding factors, so more high-quality research is needed.

## Introduction

Intracerebral hemorrhage (ICH) is the second most common cause of stroke, the incidence of which is 24.6 per 100,000 person-years, with mortality that has maintained a rate of 35.2–45.5% for several decades, which is at a much higher level compared with that of ischemic stroke (1, 2). ICH complications, such as hematoma expansion, perihematomal edema, the intraventricular extension of the hemorrhage with hydrocephalus, seizures, venous thromboembolic events, hyperglycemia, increased blood pressure, fever, and infections, can in turn increase mortality and adverse outcome after ICH onset. Of these complications, seizures are a frequent complication with an overall 30-day risk of about 8%, but their impact on ICH clinical outcomes and mortality remains to be elucidated (3).

Most previous research focused on exploring the relationship between mortality and adverse outcome and seizures after stroke, including ischemic and hemorrhagic stroke; however, the results were inconclusive on account of numerous confounding factors (4–6). As there is a higher incidence of seizures following hemorrhagic stroke compared with ischemic stroke (5–9), we performed a systematic review and meta-analysis to assess current and relevant literature to evaluate the relationship between mortality and post-ICH seizures diminutively aimed at avoiding the impact of other stroke subtypes.

## Methods

We conducted this systematic review and meta-analysis in accordance with the Preferred Reporting Items for Systematic Review and Meta-Analysis Protocols guidelines (10).

### Search Strategy

We performed systematic searches of PubMed and Embase from the establishment of the databases to December 2021 to identify literature that evaluated mortality and seizures in patients with ICH. MeSH terms, explored EMTREE headings, and keywords were applied, and the search terms included “mortality,” “seizure,” “epileptic,” and “intracerebral hemorrhage” and their variants. No language restrictions were set. We also searched references in included literature to identify additional studies. A detailed search strategy is shown in Supplementary Materials 1, 2.

### Outcomes

Our main outcome was the relationship between mortality and post-ICH seizures at the longest available follow-up. On account of the different types of post-ICH seizures, such as early seizures (ESs), late seizures (LSs), status epilepticus (SE), and any seizures (Ass), having a potential effect on mortality, subgroup analysis was used to evaluate the relationship between mortality and different types of seizures. A crude odds ratio (OR) and adjusted odds ratio (aOR), derived from a multivariable model adjusted for measured confounders, were respectively used to confirm this relationship.

### Study Selection and Data Extraction

Two researchers (HY-L and QQ-W) independently screened and evaluated the article titles and abstracts for inclusion. A third researcher (JY-H) was employed in the case of disagreements. Articles that were not relevant to the mortality of seizure onset after patients with ICH, or that merely reported the mortality of seizure onset after other stroke subtypes, such as subarachnoid hemorrhage, cerebral venous sinus thrombosis, and cerebral infarction, were excluded during the screening process. Articles that reported the relationship between mortality and seizures after a whole stroke were excluded, and articles where relevant data of ICH subtypes could not be extracted were also excluded. We also excluded duplicate records, case reports, reviews, and unavailable full text.

Diagnosis of seizures was defined as convulsive seizures or non-convulsive seizures by using an electroencephalogram (EEG) or not. According to definitions of the International League Against Epilepsy (11), seizures are generally divided into ESs and LSs, in which the former occurs within 7 days and the latter occurs after 7 days (12). ESs and LSs, as well as SE and ASs that were not able to be classified, were all included in this study. Considering that previous studies used a non-standard cut-off point for ES and LS and were not able to acquire initial data, we used an established cut-off point of each study for analysis.

Data extracted from the articles included study design, research time, sample size, the definition of seizures, effect size of the association between post-ICH seizures and mortality, and confounding factors were included in the multivariate analysis.

### Quality Assessment

Two researchers (XH-P and CX-H) independently utilized the Newcastle-Ottawa Scale (NOS) to assess each study. This scale awards a maximum of nine stars including four stars for selection of participants and measurement of exposure, two stars for comparability, and three stars for assessment of outcomes and adequacy of follow-up (13). Scores of 0 to 3, 4 to 6, and 7 to 9 were defined as low, moderate, and high-quality studies, respectively.

### Statistical Analysis

We acquired the crude ORs and aORs and their 95% confidence interval (CI) from the included studies. If the crude OR and 95% CI were not mentioned in the article, we calculated their quantitative value by the number of deaths and survivals in each of the included studies. These calculated crude ORs and aORs, and their associated 95% Cis, were then used in the meta-analysis.

The heterogeneity between the studies was quantitatively determined by the I^2^ value, which was divided into three levels: low (I^2^ = 25–49%), moderate (I^2^ = 50–74%), and high (I^2^ ≥ 25–49%). We performed subgroup analysis using categorical data to identify potential sources of heterogeneity according to the study sample size, including no previous seizure/epileptic episodes, previous seizure/epileptic episodes, and seizure type. Furthermore, we performed sensitivity analyses to explore potential sources of heterogeneity and resulting robustness by omitting one study at a time.

Publication bias was analyzed and is represented as a funnel plot. Funnel plot symmetry was assessed with Egger's test. A random-effects model, using the DerSimonian and Laird method for variance estimation, was performed in the meta-analysis (14). Statistical significance was set at a *P*-value > 0.05 (two-tailed). Stata software version 12.0 (Stata corp LP, College Station, TX, USA) was used to perform the data analysis in this meta-analysis.

## Results

### Study Selection and Characteristics

**Figure 1:**
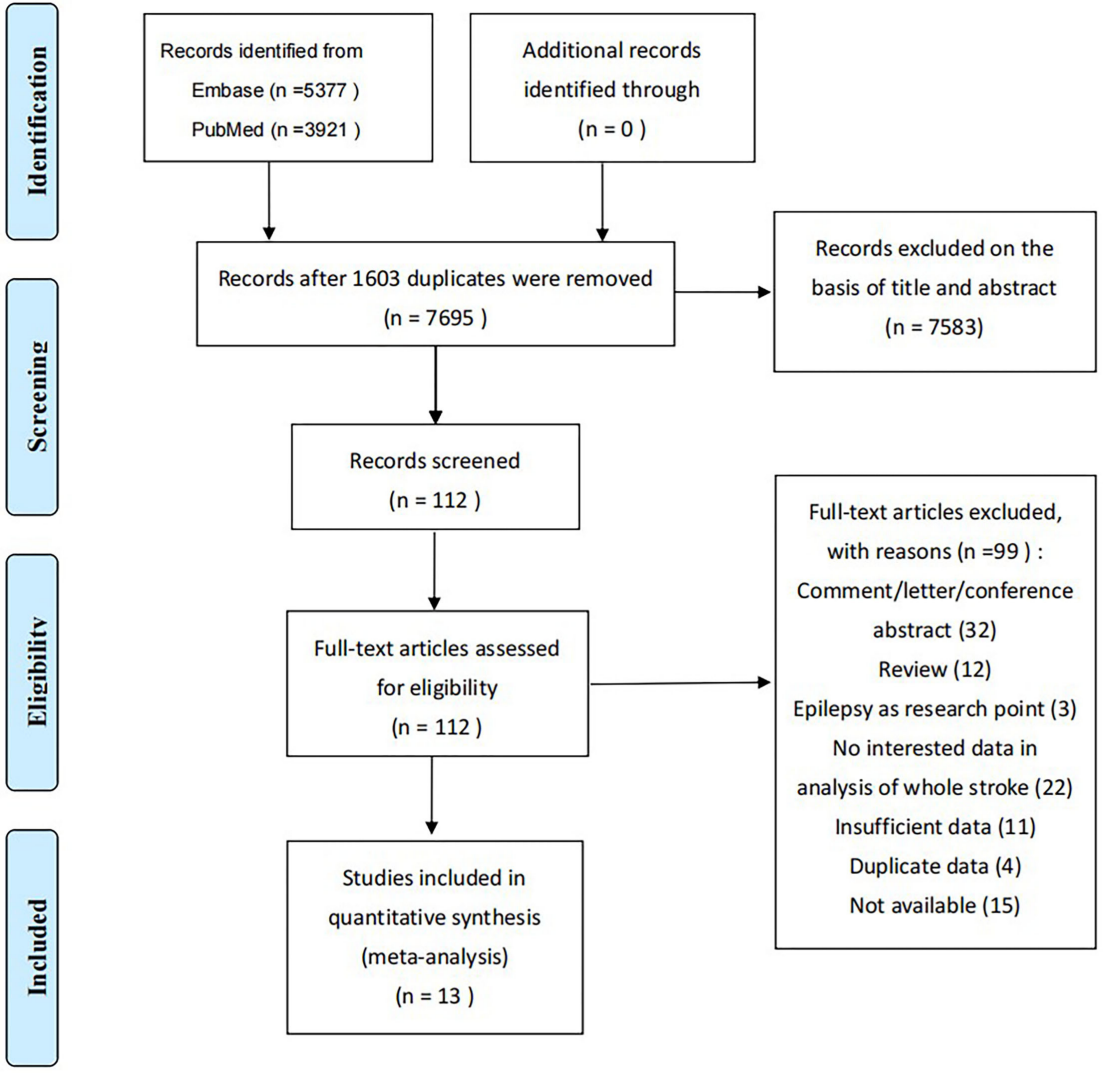
Flowchart of screening for the systematic review and meta-analysis.

There were 9,298 studies (5,377 in Embase, 3,921 in PubMed) in the primary searches for initial review; 7,695 remained after 1,603 duplicates were removed and 13 studies (15–27) were eventually included (Figure 1), in which three were retrospective and 10 prospective. A total of 245,908 patients was included and the sample size of each study differed widely with a range between 228 and 245,908. Study populations were single hospital-based, multi hospital-based, and population-based, and were from Asia, America, and Europe. Definitions of seizures included ESs, LSs, SE, and in-hospital seizures that could not be classified. ESs were defined as seizures occurring within 7, 14, or 30 days after ICH onset.

Three included studies (17, 24, 26) provided crude ORs and 95% CIs directly, two others (16, 25) provided no related data on crude ORs, and the remaining eight studies (15, 18–23, 27) provided the numbers of deaths and survivals of seizure and non-seizure groups, which were then used to calculate the crude ORs and 95% CIs as described in the methods. Among them, Claessens et al. (22) provided the number of deaths and survivals associated with ESs and LSs separately; we, therefore, analyzed the two as a whole seizure in the main outcome and analyzed each one respectively in the subgroup analysis. Only six included studies (16, 17, 21, 23–26) provided aORs and 95% CIs. Detailed information is presented in Table 1.

**Table 1:** Characteristic of included studies.

**Study**	**Design**	**Country**	**Research time**	**Population**	**Age**	**Diagnostic mode of ICH**	**Sample size**	**Including previous seizures/ epilepsy**	**Endpoint time of mortality**	**Definition of seizures**	**Death of seizures group**				**Death of non-seizures group**	**crude OR (95%CI)**	**aOR (95%CI)**	**Confounders**
											**ES**	**LS**	**SE**	**AS**				
Burneo et al. (15)	Prospective	Canada	July 2003 to June 2005	Multi hospital-based	NR	NR	939	NR	1 year	In-hospital	-	-	-	27/54	419/885	1.11 (0.64 - 1.93)	-	-
Zöllner et al. (16)	Prospective	Germany	January 2004 to December 2016	Multi hospital-based	≥18 years old	ICD-10: I61.x	15,928	NR	at discharge	In-hospital	-	-	-	NR	NR	-	0.70 (0.55 - 0.90)	Age, sex, and GCS score <13
Li et al. (17)	Prospective	China	September 2007 to August 2008	national population-based	NR	according to World Health Organization criteria	3,216	no	1 year	In-hospital	-	-	-	NR	NR	2.90 (2.06 - 4.08)	1.97 (1.27 - 3.05)	Age, sex, smoking, heavy drinking, history of stroke, hypertension, diabetes mellitus, dyslipidemia, cardiovascular disease, atrial fibrillation, National Institutes of Health Stroke Scale score and GCS score on admission, hematoma volume, hematoma location, intraventricular hemorrhagic extension, in-hospital complications including hematoma expansion, atrial fibrillation, urinary tract infection, decubitus ulcer, myocardial infarction, deep venous thrombosis, pneumonia, and gastrointestinal bleeding and performance measures of antihypertensive therapy, dysphagia screening, and rehabilitation evaluation
Liao et al. (18)	Prospective	Taiwan	January 2006 to December 2009	single hospital-based	NR	NR	297	NR	in-hospital mortality	ES (<7 days)	5/9	-	-	-	66/288	4.21 (1.10 - 16.11)	-	-
Brüning et al. (19)	Prospective	Germany	2009 to 2013	single hospital-based	NR	NR	461	yes	in-hospital mortality	ES (<7 days)	5/52	-	-	-	98/409	0.34 (0.13 - 0.87)	-	-
Herdt et al. (20)	Prospective	France	November 2004 to March 2009	single hospital-based	NR	primary ICH	508	no	6 months	ES (<7 days)	32/71	-	-	-	235/437	0.71 (0.43 - 1.17)	-	-
Law et al. (21)	Prospective	international	March 2013 to September 2017	multi hospital-based	>18 years	spontaneous ICH	2,101	NR	90 days	ES (<7 days) LS (≥7 days)	38/139	-	-	-	237/1,962	2.74 (1.84 - 4.07)	3.26 (1.98 - 5.39)	Age, sex, premorbid modified Rankin Scale, prior antiplatelet therapy, National Institute of Health Stroke Scale, systolic blood pressure, onset to CT <3 h, baseline haematoma volume, intraventricular hemorrhage and lobar location
Claessens et al. (22)	Retrospective	the Netherlands	January 2004 to December 2009	multi hospital-based	>18 years	non-traumatic ICH	747	no	up to 10 years	ES (< 7 days) LS (≥ 7 days)	20/32	45/74	-	-	400/641	0.96 (0.63 - 1.46)	-	-
Matsubara et al. (23)	Retrospective	Japan	August 2014 to July 2016	single hospital-based	NR	non-traumatic ICH	228	no	at discharge	in-hospital NCSE	-	-	6/20	-	21/208	3.82 (1.33 - 10.99)	2.3 (0.7 - 7.0)	Sex and the ICH score
Hamidou et al. (24)	Prospective	France	January 1985 to December 2010	population-based	NR	according to World Health Organization criteria	493	NR	1 year	ES (<14 days)	?/31	-	-	-	?/462	0.86 (0.49 - 1.52)	0.66 (0.34 - 1.19)	Gender, age, stroke subtypes, history of hypertension, hypercholesterolemia, heart failure, smoking status, atrial fibrillation, myocardial infarction, motor deficit, sensory deficit, aphasia, impaired consciousness, blood glucose at admission, antihypertensive drugs, and anticoagulants
Mehta et al. (25)	Retrospective	United States	1999 to 2011	population-based	NR	ICD-9-CM: 431, 432.9	220,075	NR	in-hospital mortality	ICD-9-CM: 780.3, 780.31, 780.39	-	-	-	?/26,237	?/193,838	-	0.75 (0.72 - 0.77)	NR
Passero et al. (26)	Prospective	Italy	January 1979 and December 1996	single hospital-based	NR	non-traumatic nonaneurysmal ICH	650	no	in-hospital mortality	ES (<30 days)	16/25	-	-	-	?/625	1.01 (0.77 - 1.33)	-	-
Bladin et al. (27)	Prospective	international	NR (34 months of follow-up)	multi hospital-based	NR	primary ICH	265	no	1 year	ES (<14 days)	-	-	-	13/28	85/237	1.55 (0.70 - 3.41)	-	-

*ICH indicates intracerebral hemorrhage; ES, early seizures; LS, late seizures; SE, status epilepticus; AS, any seizures; OR, odds ratio; aOR, adjusted odds ratio; CI, confidence interval; GCS, Glasgow Coma Scale; NCSE, non-convulsive status epilepticus; NR, not reported; CT, computed tomography; and ICD-9-CM, International Classification of Diseases-Ninth Revision-Clinical Modification. ? means irretrievable data from original study; - means no relevant data*.

### Study Quality

Two studies received nine stars after NOS quality assessment, seven received eight stars, three received seven stars, and one received six stars, which, taken together, indicated moderate to high-quality studies (Table 2).

**Table 2:** Methodological quality assessment of included studies by new castle-ottawa scales.

**Study**	**Selection**				**Comparability**		**Outcome**		**Total score**
	**Exposed cohort**	**Non-exposed cohort**	**Ascertainment of exposure**	**Outcome of interest**		**Assessment of outcome**	**Length of follow-up**	**Adequacy of follow-up**	
Burneo et al. (15)	*	*	*	*	*	*	*	*	8
Zöllner et al. (16)	*	*	*	*	**	*	—	*	8
Li et al. (17)	*	*	*	*	**	*	*	*	9
Liao et al. (18)	*	*	*	*	*	*	—	*	7
Brüning et al. (19)	*	*	*	—	*	*	—	*	6
Herdt et al. (20)	*	*	*	*	**	*	—	*	8
Law et al. (21)	*	*	*	*	**	*	—	*	7
Claessens et al. (22)	*	*	*	*	*	*	*	*	8
Matsubara et al. (23)	*	*	*	*	**	*	—	*	8
Hamidou et al. (24)	*	*	*	*	**	*	*	*	9
Mehta et al. (25)	*	*	*	*	**	*	—	*	8
Passero et al. (26)	*	*	*	*	*	*	—	*	7
Bladin et al. (27)	*	*	*	*	*	*	*	*	8

*Single asterisk indicates 1 score, double asterisk indicates 2 scores, and dash indicates 0 scores*.

### Main Outcome

**Figure 2:**
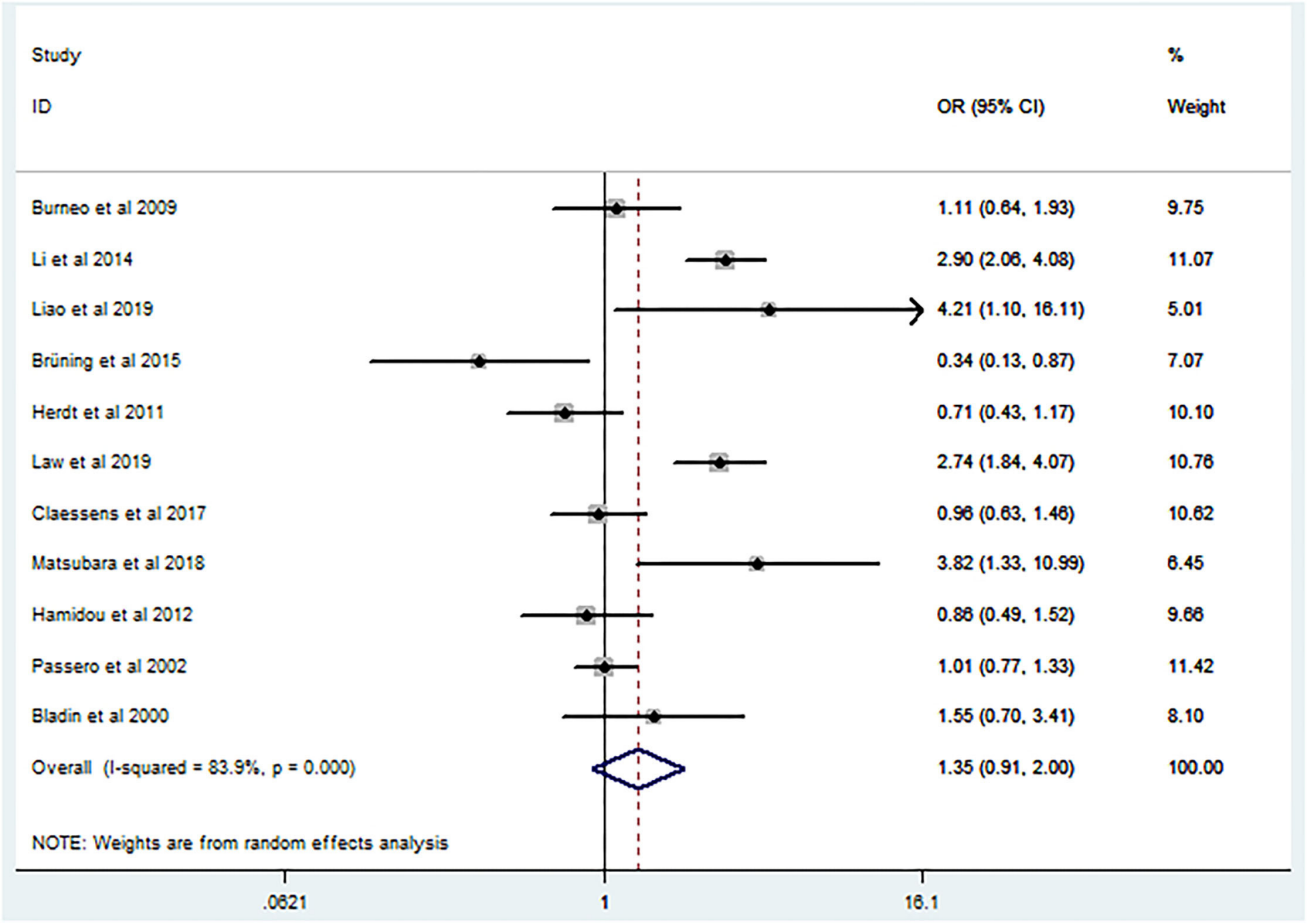
Forest plot of crude odds ratio on the relationship between mortality and seizures after intracerebral hemorrhage.

**Figure 3:**
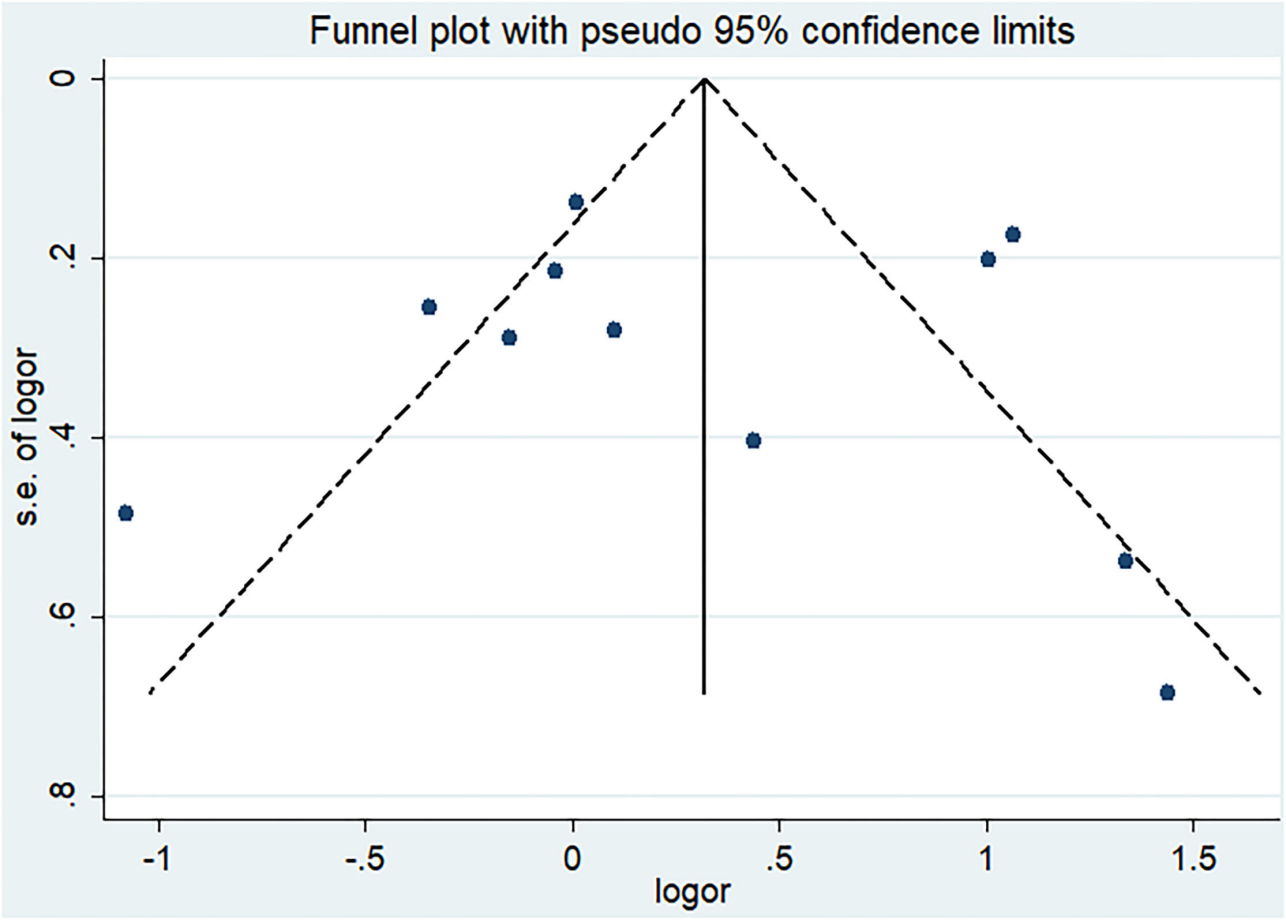
Funnel plot for publication bias of the relationship between mortality and seizures after intracerebral hemorrhage.

Post-ICH seizures were not associated with significantly increased mortality in patients with ICH (crude OR 1.35, 95% CI 0.91–2; Figure 2). The *p*-value for the statistic was <0.001, which suggested that the true effect sizes did not vary among the studies included in the meta-analysis. The I^2^ statistic was 83.9%, which demonstrated a high heterogeneity, suggesting that 83.9% of the variance in the observed effects was due to differences among the true effect sizes and not sampling errors. Publication bias analysis did not highlight any differences between observed and estimated values (Figure 3). Egger's test was not statistically significant (*p* = 0.95).

**Figure 4:**
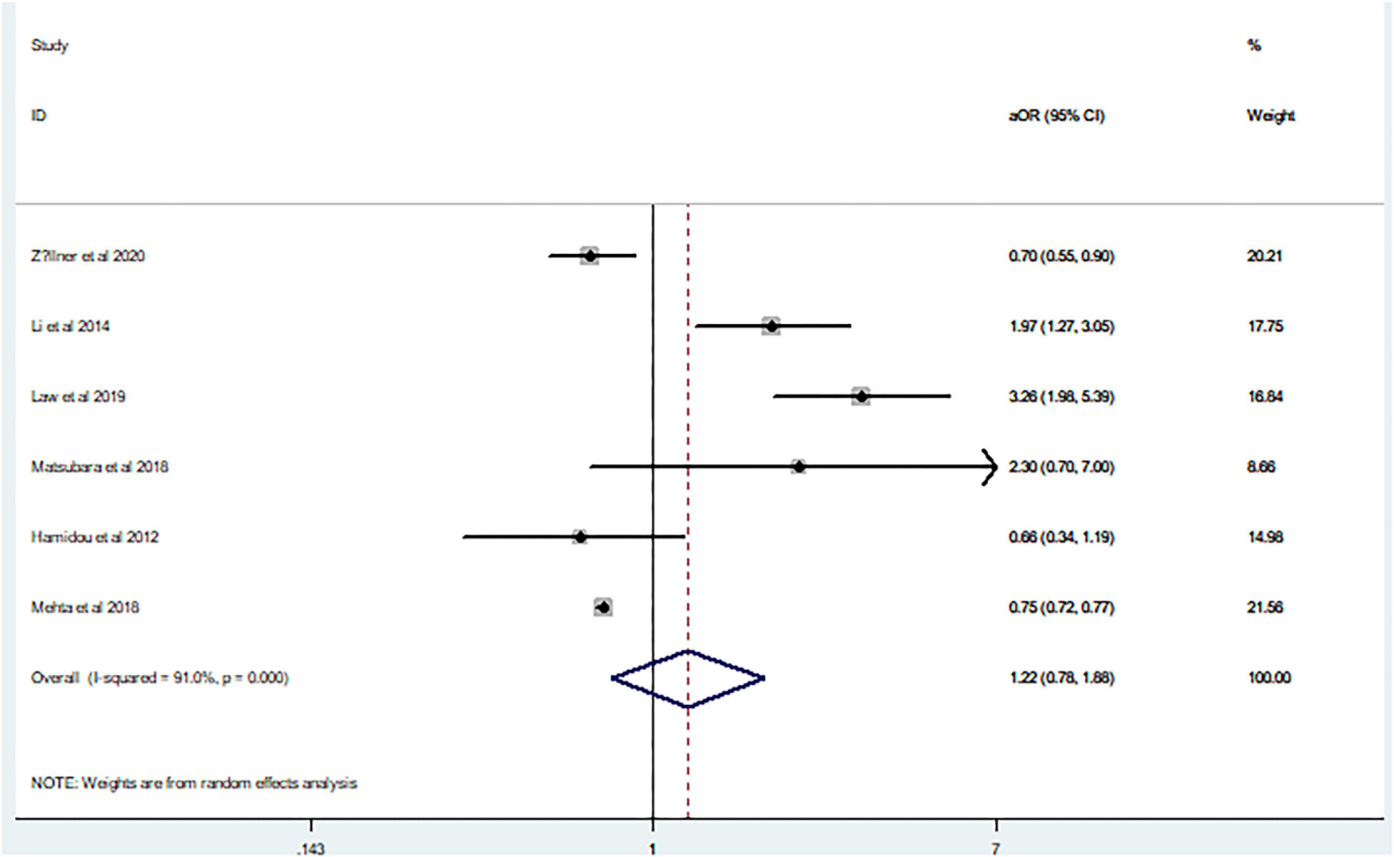
Forest plot of the adjusted odds ratio on the relationship between mortality and seizures after intracerebral hemorrhage.

In agreement with the above results, aOR (aOR 1.22, 95% CI 0.78–1.88) also showed a high heterogeneity (I^2^ = 91%, *p* = 0.00; Figure 4). Because only six studies were included in the aOR meta-analysis, funnel plot asymmetry was not conducted given the limited specificity and power of these tests when fewer than ten studies are included.

### Subgroup Analysis

We performed subgroup analysis on the study sample size, including no previous seizures/epileptic episodes, previous seizures/epileptic episodes, and seizure type. The results showed that the crude OR in the subgroup of the sample size was ≥ 1,000, with previous seizures/epileptic episodes and SE showing a positive relationship, whereas aOR in the subgroup of no previous seizures/epileptic episodes showed a positive relationship (Table 3).

**Table 3:** Subgroup analysis of crude OR and ajusted OR.

**Sub-group analysis**	**Classification**	**OR**	**aOR**
Sample size	<500	1.37 (0.61–3.08) (18, 19, 24, 25, 27)	1.12 (0.33–3.74) (23, 24)
	≥500– <1,000	0.96 (0.77 - 1.19) (15, 20, 26)	-
	≥ 1,000	1.98 (1.01 - 3.90) (17, 21, 22)	1.28 (0.76 - 2.15) (16, 17, 21, 25)
Including previous seizures/epilepsy	Yes	0.34 (0.13–0.87) (19)	-
	No	1.42 (0.85–2.36) (17, 20, 22, 23, 26, 27)	2.01 (1.33–3.03) (17, 23)
	No report	1.66 (0.83–3.29) (15, 18, 21, 24)	1.00 (0.63–1.57) (16, 21, 24, 25)
Seizures type	ES	1.09 (0.67–1.79) (18–22, 24, 26)	1.48 (0.31–7.10) (21, 24)
	LS	0.94 (0.57–1.53) (22)	-
	SE	3.82 (1.33–10.99) (23)	2.30 (0.70–7.00) (23)
	AS	1.77 (0.91–3.45) (15, 17, 27)	0.95 (0.64 - 1.41) (16, 17, 25)

*OR, odds ratio; aOR, adjusted odds ratio. - means no relevant data*.

### Sensitivity Analysis

When the study of Brüning et al. was omitted for meta-analysis, there was a slight positive relationship between post-ICH seizures and mortality (OR 1.49, 95% CI 1.01–2.20). When other studies were omitted in turn, this relationship became non-significant (Table 4).

**Table 4:** Sensitivity analysis of crude OR.

**Study omitted**	**Crude OR**	**95% CI**
Burneo et al. (15)	1.38	0.90–2.13
Li et al. (17)	1.22	0.84–1.77
Liao et al. (18)	1.27	0.85–1.90
Brüning et al. (19)	1.49	1.01–2.20
Herdt et al. (20)	1.45	0.96–2.19
Law et al. (21)	1.24	0.83–1.85
Claessens et al. (22)	1.41	0.91–2.18
Matsubara et al. (23)	1.26	0.84–1.88
Hamidou et al. (24)	1.42	0.93–2.16
Passero et al. (26)	1.41	0.90–2.21
Bladin et al. (27)	1.34	0.88–2.03

In the sensitivity analysis of aOR, there was no relationship between post-ICH seizures and mortality when each study was omitted (Table 5). Hence, our results were relatively robust in the sensitivity analysis.

**Table 5:** Sensitivity analysis of adjusted OR.

**Study omitted**	**aOR**	**95% CI**
Zöllner et al. (16)	1.44	0.72–2.88
Li et al. (17)	1.08	0.69–1.68
Law et al. (21)	0.95	0.67–1.34
Matsubara et al. (23)	1.14	0.73–1.80
Hamidou et al. (24)	1.36	0.83–2.24
Mehta et al. (25)	1.42	0.70–2.88

## Discussion

This meta-analysis explored the relationship between seizures and mortality in patients with ICH, the results of which indicated that no relationship was evident. That is, post-ICH seizures were not associated with significantly increased mortality in patients with ICH.

However, there were interactions between seizures and ICH. ESs could be caused by mechanical effects of the expanding hemorrhage, the disruption of cortical networks by hematoma via its structural damaging properties, and/or irritation of the cortex due to products of blood metabolism (28). LSs are thought to be related to gliosis and chemical-cellular repair processes creating an epileptogenic focus (7, 8, 29). Seizures could in turn increase the severity and mortality since they occur after ICH. Early epileptiform activity has a negative impact on perihematomal areas, possibly by increasing the cerebral blood flow and glucose metabolic demand in hypoxic tissue and may also be related to the molecular pathophysiology associated with the activation of cytotoxic, oxidative, and inflammatory pathways that result in surrounding cells death (30–32). Additionally, symptomatic seizures due to stroke or other diseases resulted in increased early mortality rates (33).

To our surprise, only four included studies corresponded with the above theory (17, 18, 21, 23). among which Matsubara et al. (23) supported a positive result only in the crude analysis, but not in the adjusted analysis. Six included studies (15, 20, 22, 24, 26, 27), found no relationship between seizures and ICH, while the remaining three (16, 19, 25) found that post-ICH seizures were associated with reduced odds of mortality. The cause of the reduced odds of mortality reduction was related to the fact that seizure patients had less severe neurologic injuries, and more severely injured patients may be more likely to suffer non-convulsive seizures that would be underdiagnosed without EEG, and seizures patients tended to receive intensive care once seizures occurred (16, 19, 25).

Our results inferred those seizures were not associated with significantly increased mortality in patients with ICH, which was consistent with the analysis of crude OR (1.35, 95% CI 0.91–2) and aOR (1.22, 95% CI 0.78–1.88). This result was relatively robust in the sensitivity analysis but could be influenced by some potential risk factors. Furthermore, the sample sizes of the included studies may have influenced the robustness of the results. Overall, as the findings indicated that the occurrence of seizures in patients with preexisting epilepsy tended to be higher than in those without preexisting epilepsy (19), mortality rates may be different between patients with preexisting epilepsy and those without. In addition, the mechanism of different types of post-ICH seizures was not dissimilar and their impact on ICH may have led to discrepancies in the data. Because of these three potential sources of heterogeneity, we conducted a subgroup analysis. We found that a positive relationship tended to appear as the sample sizes increased. Inclusion of no previous seizures/epileptic episodes and previous seizures/epileptic episodes may have led to inconsistent results and which are considered potential confounding factors. Type of seizure had no effect on increased mortality in patients with ICH, except SE. However, only one study provided data on SE.

There is controversy surrounding whether patients should receive antiepileptic drugs (AEDs) as primary pharmacological prevention of seizures after spontaneous intracerebral hemorrhage (34). The use of AEDs depends on a balance of the effect of seizures on prognosis of patients with ICH, the effect of AEDs on reducing incidence of post-ICH seizures and mortality of patients with ICH after the occurrence of seizures, and the toxicity and side effects of AEDs. A recent meta-analysis inferred that the use of AEDs as primary prevention among adult patients with spontaneous intracerebral hemorrhage was not associated with improved neurological function during long-term follow-up (34). Moreover, according to European Stroke Organization guidelines for the management of post-stroke seizures and epilepsy, little evidence exists for the recommendation of primary seizure prophylaxis for ICH (35). Hence, our results that no association was found between post-ICH seizures and mortality support these recommendations.

Our study had several limitations. First, included studies were all observational studies and many potential confounding factors likely remained. Second, the cut-off points of ES and LS in included studies were inconsistent, most of which was defined as 7 days; however, other was extended to 14 days or even 30 days from stroke onset. The incidence rate of ES and LS could be influenced by the inconsistent cut-off point and could eventually influence mortality. Third, most included studies did not have a standard method of seizure monitoring and relied on clinical detection of seizures, which has resulted in under-representation of non-convulsive seizures that may have impacted clinical outcomes. Fourth, follow-up times of the included studies varied as the mortality rates may increase with increasing time. Fifth, most studies did not provide data on AEDs usage, which may affect the occurrence of post-ICH seizures and further weaken its effect on the outcome of ICH. Finally, our results were not consistent in all subgroup analyses, which indicated that the results could be influenced by many factors.

Conversely, our study had several strengths. It is the first meta-analysis published to date on the relationship of mortality and seizures after ICH. Furthermore, we explored this relationship by using crude ORs and aORs, respectively, and the results were found to be consistent. Finally, we conducted subgroup and sensitivity analyses and the results were found to be relatively robust relatively in the sensitivity analysis.

## Conclusions

In conclusion, the available evidence revealed that post-ICH seizures were not associated with a significant increased mortality in patients with ICH. However, the results of the current study may be influenced by confounding factors and, therefore, more high-quality research is needed.

## Data Availability Statement

The original contributions presented in the study are included in the article/Supplementary Material, further inquiries can be directed to the corresponding author/s.

## Author Contributions

H-yL and Q-qW: conceptualization, literature search, and original draft. J-yH and WG: methodology. X-hP and C-xH: data collection. TL and N-cL: data analysis. S-lS: review, editing, and supervision. All authors contributed to the article and approved the submitted version.

## Conflict of Interest

The authors declare that the research was conducted in the absence of any commercial or financial relationships that could be construed as a potential conflict of interest.

## Publisher's Note

All claims expressed in this article are solely those of the authors and do not necessarily represent those of their affiliated organizations, or those of the publisher, the editors and the reviewers. Any product that may be evaluated in this article, or claim that may be made by its manufacturer, is not guaranteed or endorsed by the publisher.

## Abbreviations

ICH: intracerebral hemorrhage
ES: early seizures
LS: late seizures
SE: status epilepticus
AS: any seizures
OR: odds ratio
aOR: adjusted odds ratio
EEG: electroencephalogram
NOS: Newcastle-Ottawa Scale
CI: confidence interval
AEDs: antiepileptic drugs
GCS: Glasgow Coma Scale
NCSE: nonconvulsive status epilepticus
ICD-9-CM: International Classification of Diseases-Ninth Revision-Clinical Modification
CT: computed tomography
NR: not reported.
