# Supplementary material for: Relationship Between Mortality and Seizures After Intracerebral Hemorrhage: A Systematic Review and Meta-Analysis
Source: Front Neurol. 2022 Jun 20;13:922677. doi: 10.3389/fneur.2022.922677 (PMC9251061; doi:10.3389/fneur.2022.922677)
Supplement: Supplementary file 1 [file Table_1.pdf]

Source: Embase; Search on: November 30, 2021; Result: 5377

Search	Query
1	brain hemorrhage'/exp
2	brain hemorrhage':ab,ti
3	brain hemorrhages':ab,ti
4	cerebral hemorrhage':ab,ti
5	cerebral hemorrhages':ab,ti
6	intracranial hemorrhage':ab,ti
7	intracranial hemorrhages':ab,ti
8	intracerebral hemorrhage':ab,ti
9	intracerebral hemorrhages':ab,ti
10	cerebral:ab,ti OR intracerebral:ab,ti OR intracranial:ab,ti OR brain:ab,ti OR hypertensive:ab,ti
11	hemorrhag*:ab,ti OR haemorrhag*:ab,ti OR hematoma:ab,ti OR bleed*:ab,ti
12	#10 AND #11
13	hemorrhag*:ab,ti OR haemorrhag*:ab,ti
14	cerebrovascular disease':ab,ti OR 'cerebrovascular diseases':ab,ti OR 'cerebral vascular disease':ab,ti OR 'cerebral vascular diseases':ab,ti OR stroke:ab,ti OR strokes:ab,ti
15	#13 AND #14
16	hematencephalon:ab,ti
17	encephalorrhagia:ab,ti
18	#1 OR #2 OR #3 OR #4 OR #5 OR #6 OR #7 OR #8 OR #9 OR #12 OR #15 OR #16 OR #17
19	seizure'/exp
20	seizure:ab,ti
21	seizures:ab,ti
22	convulsi*:ab,ti
23	epilepsy'/exp
24	epilep*:ab,ti
25	#19 OR #20 OR #21 OR #22 OR #23 OR #24
26	mortality'/exp
27	mortality:ab,ti
28	mortalities:ab,ti
29	survival:ab,ti
30	survivals:ab,ti
31	death:ab,ti
32	deaths:ab,ti
33	#26 OR #27 OR #28 OR #29 OR #30 OR #31 OR #32

34	#18 AND #25 AND #33

Results
161,934
1,365
213
6,956
662
17,201
1,570
19,936
1,299
1,983,348
732,709
139,301
381,647
463,310
52,227
12
25
226,536
165,462
107,336
149,186
36,749
269,035
216,537
431,849
1,230,647
1,259,885
15,594
1,548,933
16,043
1,107,738
285,572
3,697,294

5,377
