# Supplementary material for: Relationship Between Mortality and Seizures After Intracerebral Hemorrhage: A Systematic Review and Meta-Analysis
Source: Front Neurol. 2022 Jun 20;13:922677. doi: 10.3389/fneur.2022.922677 (PMC9251061; doi:10.3389/fneur.2022.922677)
Supplement: Supplementary file 2 [file Table_2.pdf]

Source: PubMed; Search on: November 30, 2021; Result: 3,921

Search	Query
1	cerebral hemorrhage[MeSH]
2	cerebral hemorrhage[tiab]
3	cerebral hemorrhages[tiab]
4	intracranial hemorrhages[MeSH]
5	intracranial hemorrhage[tiab]
6	intracranial hemorrhages[tiab]
7	intracerebral hemorrhage[tiab]
8	intracerebral hemorrhages[tiab]
9	brain hemorrhage[tiab]
10	brain hemorrhages[tiab]
11	(cerebral or intracerebral or intracranial or brain or hypertensive) AND (hemorrhag* or haemorrhag* or hematoma or bleed*)
12	hemorrhag* or haemorrhag*
13	(((((cerebrovascular disease) OR (cerebrovascular diseases)) OR (cerebral vascular disease)) OR (cerebral vascular diseases)) OR (stroke)) OR (strokes)
14	#12 AND #13
15	hematencephalon[tiab]
16	encephalorrhagia[tiab]
17	#1 OR #2 OR #3 OR #4 OR #5 OR #6 OR #7 OR #8 OR #9 OR #10 OR #11 OR #14 OR #15 OR #16
18	seizures[MeSH]
19	seizure [tiab]
20	seizures [tiab]
21	convulsi*[tiab]
22	epilepsy[MeSH]
23	epilep* [tiab]
24	#18 OR #19 OR #20 OR #21 OR #22 OR #23
25	mortality[MeSH]
26	mortality[tiab]
27	mortalities[tiab]
28	survival[tiab]
29	survivals[tiab]
30	death[tiab]
31	deaths[tiab]
32	#25 OR #26 OR #27 OR #28 OR #29 OR #30 OR #31
33	#17 AND #24 AND #32

Results
35,674
7,595
477
75,525
11,110
1,218
13,677
925
1,028
155
766,399
404,762
669,323
105,220
8
12
786,132
67,967
71,092
99,656
27,703
118,370
151,707
255,995
410,719
865,699
11,242
1,050,655
10,048
789,223
198,382
2,511,337
3,921
